# Projected burden of hypertension-associated cardiovascular disease in people living with HIV versus HIV-negative adults in Eswatini

**DOI:** 10.64898/2026.07.17.26358301

**Published:** 2026-07-20

**Authors:** Masabho P. Milali, Daniel T. Citron, Kasturi Bhamidipati, Nao Yamamoto, Afia Osei-Ntansah, Ingrida Platais, Gianna Ferrara, Cebisile Ngcamphalala, Sotja G. Dlamini, Ntombifuthi Ginindza, Anna Bershteyn

**Affiliations:** 1 Department of Population Health, NYU Grossman School of Medicine, New York, NY, USA; 2 Department of Agricultural Economics and Management, Faculty of Agriculture, University of Eswatini; 3 Ministry of Health in Eswatini, Ministry of Justice & Constitutional Affairs Building, Mhlambanyatsi Road, Mbabane, Eswatini.

**Keywords:** Cardiovascular disease, People living with HIV, Hypertension, Forecasting, Dolutegravir, Mathematical modeling, Eswatini

## Abstract

**Background:**

Having achieved the UNAIDS 95–95–95 targets, Eswatini faces a growing burden of non-communicable diseases which are major contributors to morbidity and mortality. Hypertension-associated cardiovascular disease (CVD) is rising among people living with HIV (PLHIV) as survival improves and metabolic risks – including those linked to dolutegravir (DTG) – increase. We projected CVD burden among PLHIV and HIV-negative adults (PLWHIV) through 2045 to inform integrated HIV–CVD planning.

**Methods:**

EMOD-HIV, an agent-based model calibrated to Eswatini’s epidemic, generated HIV prevalence trajectories. These were combined with age-standardized Global Burden of Disease CVD estimates and published relative risks (RRs) to produce HIV-stratified CVD projections. CVD burden trajectories were then projected through 2045 using a logistic generalized additive model with Monte Carlo uncertainty quantification. Five scenarios were evaluated to assess how different assumptions about RR of CVD among PLHIV versus PLWHIV affect projected burden: (1) CVD prevalence under a constant RR; (2) CVD mortality under a constant RR; (3) HTN-attributable CVD mortality under a constant RR; (4) HTN-attributable CVD mortality under a post-DTG RR increase following Eswatini’s 2021 dolutegravir rollout; and (5) HTN-attributable CVD mortality under a gradual RR increase from 2010–2045 reflecting cumulative metabolic and demographic shifts.

**Results:**

PLHIV consistently exhibited higher CVD burden than HIV-negative adults. Scenario 1: CVD prevalence was 11.0% (95% UI: 9.0–13.5%) among PLHIV versus 6.8% (6.0–7.8%) in 2025, stable through 2045. Scenario 2: CVD mortality rate was 0.60% (0.47–0.76%) versus 0.37% (0.31–0.45%) in 2025, declining modestly through 2045 with consistent excess. Scenario 3: HTN-attributable mortality was 73% (70–76%) versus 64% (61–66%) in women and 61% (58–64%) versus 58% (55–60%) in men, stable through 2045. Scenario 4: Following DTG rollout, mortality rose from 73% to 85% in women and 61% to 70% in men by 2022, remaining stable thereafter. Scenario 5: By 2045, mortality reached 85% (82–88%) in women and 67% (64–70%) in men with HIV, versus 60% (57 – 63%) and 55% (53 – 57%) in HIV-negative adults.

**Conclusions:**

While excess CVD burden among PLHIV is projected to persist even under stable risk conditions, ART-related metabolic trajectories – particularly those linked to DTG – may drive substantial widening of this gap through 2045. HTN-attributable CVD mortality is particularly elevated among women with HIV. Strengthening integrated HIV–NCD services, including blood pressure screening, risk-based therapy, and sex-specific DTG counseling, will be essential to sustain long-term health gains.

## Introduction

Cardiovascular disease (CVD) has emerged as a leading cause of morbidity and mortality in sub-Saharan Africa, accounting for nearly one-third of all non-communicable disease (NCD) deaths (1,2). In Eswatini, CVD risk is compounded by the world’s highest HIV prevalence—approximately 25% among adults aged 15–49 years (3–5), and the country’s rapid epidemiologic transition toward NCDs (6). As people living with HIV (PLHIV) experience longer survival under antiretroviral therapy (ART), comorbidities such as hypertension, diabetes, and dyslipidemia have become increasingly common (3,7). In settings with high ART coverage and viral suppression, these chronic conditions contribute an increasing share of morbidity and mortality among adults with HIV (8).

Growing evidence links both HIV infection and ART exposure to elevated cardiometabolic risk through mechanisms including chronic immune activation, endothelial dysfunction, and metabolic effects of modern antiretroviral regimens (7,9–12). Notably, dolutegravir (DTG)–based ART, now first-line in Eswatini’s national program, has been associated with accelerated weight gain and increased risk of hypertension, particularly among women (9,10,13,14). As the country expands DTG use, these metabolic shifts may interact with population aging and rising background hypertension prevalence, amplifying the CVD burden among PLHIV.

Eswatini has made substantial progress in integrating NCD services within HIV care, including routine blood pressure screening, diabetes and lipid testing, and lifestyle counseling (5). However, the long-term implications of ART-related metabolic changes for national CVD trends among PLHIV in Eswatini remain unclear. Few studies in Eswatini address DTG-associated weight gain (3,14,16) and implementation of CVD risk screening within HIV services. (15,17) To date, no studies have attempted to quantify how evolving ART patterns, population aging, and secular trends in adiposity and hypertension will jointly shape future CVD burden over the next decades.

To address this gap, we developed demographic and HIV epidemiological projections using the agent-based Epidemiological MODeling–HIV (EMOD-HIV) platform (18,19), calibrated to Eswatini’s HIV epidemic, and integrated it with hypertension and CVD parameters derived from the Global Burden of Disease (GBD) (20) and WHO STEPwise (STEPS) surveys (21). We simulated CVD- and hypertension-attributable mortality through 2045 under alternative relative-risk (RR) scenarios reflecting key epidemiologic transitions, including ART-related metabolic effects, survival-driven population aging among PLHIV, and secular increases in adiposity and hypertension. We hypothesized that these interacting transitions would lead to a persistently higher—and potentially widening—CVD burden among people living with HIV compared with HIV-negative adults over time. The aim of this study was to project and compare long-term CVD burden by HIV status under plausible future risk trajectories to inform integrated HIV–NCD planning in Eswatini.

## Methods

### Ethics approval and consent to participate

This study did not involve human participants, human specimens, or identifiable individual-level data. The analysis was based on mathematical modeling using publicly available, aggregated data from the Global Burden of Disease (GBD) study, World Health Organization STEPwise (STEPS) surveys, published literature, and outputs from a previously developed and calibrated EMOD-HIV simulation model. Therefore, institutional ethics approval and informed consent were not required.

### Study overview

This modeling study used the Epidemiological MODeling–HIV (EMOD-HIV) agent-based model (18,19) to forecast the burden of hypertension (HTN)-associated cardiovascular disease (CVD) in Eswatini over a 20-year horizon (2025 to 2045). HTN-associated CVD was defined as acute myocardial infarction (AMI/heart attack), stroke (ischemic or hemorrhagic), or heart failure. EMOD-HIV was first calibrated to replicate Eswatini’s population structure and HIV epidemic dynamics using data from the 2006–7 Demographic and Health Survey (DHS) (22,23) and the Swaziland HIV Incidence Measurement Surveys (SHIMS) conducted 2011, 2016–17, and 2021 (24,25). Because EMOD-HIV does not natively simulate non-communicable diseases, CVD parameters were incorporated in a post-processing step outside the model, using age-standardized estimates obtained directly from the Global Burden of Disease (GBD) study (20). Three related but conceptually distinct outcomes were derived from these GBD datasets: (1) CVD prevalence – the proportion of the total adult population living with CVD in a given year (denominator: total population); (2) CVD mortality rate – the annual risk of dying from CVD, expressed as deaths per 100,000 person-years (denominator: total population); and (3) the proportion of CVD deaths attributable to elevated systolic blood pressure – the hypertension-mediated share of CVD mortality (denominator: total CVD deaths, not total population). These metrics are complementary but not directly comparable, as they express burden relative to different reference groups. These age-standardized CVD prevalence estimates were then combined with age- and sex-specific HIV prevalence trajectories generated by EMOD-HIV to reconstruct HIV-negative and HIV-positive CVD prevalence time series for the Eswatini adult population. Population-level prevalence was obtained by weighting HIV-stratified prevalence by the corresponding HIV prevalence in each year. Projections were stratified by HIV status, enabling direct comparisons between people living with HIV (PLHIV) and HIV-negative individuals.

### Model description

EMOD-HIV is an agent-based simulation framework developed by the Institute for Disease Modeling at the Gates Foundation and made publicly available as open-source software (19,26). It captures both heterosexual and vertical transmission, HIV disease progression, and transitions along the HIV prevention and care continuum (19,26). Sexual relationships are modeled explicitly (long-term, informal, short-term, and transactional), forming and dissolving dynamically based on demographic and behavioral characteristics. Transmission risks are modeled per coital act and at birth, with modifications reflecting prevention and treatment uptake that alter infectiousness or susceptibility (19,26). EMOD has been extensively validated and applied in multiple African settings to assess HIV prevention and treatment strategies (27–29).

### Model calibration

EMOD-HIV was calibrated to Eswatini using SHIMS (24) and DHS (22) data. Calibration employed parallel simultaneous perturbation optimization, a stochastic gradient descent algorithm adapted for parallel computing (30,31), to tune uncertain parameters related to epidemic initialization and sexual behavior. Targets included population age and sex structure (from census data), age- and sex-specific HIV prevalence and incidence (from SHIMS and DHS), and HIV service coverage (from national health agencies). The timeline of HIV care was parameterized based on SHIMS and DHS data, tracking the expanded distribution of ART, voluntary medical male circumcision (VMMC), and antenatal care. The distribution of PrEP was parameterized using data from PrEPWatch (32). We generated 250 calibrated epidemic trajectories using a likelihood-weighted roulette sampling approach (33), representing alternative but plausible epidemic histories consistent with observed data. These trajectories formed the basis for estimating age- and sex-specific HIV prevalence over time. Model performance was validated against SHIMS data from 2007, 2011, 2016, and 2021 (Supplementary Figures S1–S2).

### Integration of CVD and HTN-associated CVD parameters

This section describes the data sources, estimation and forecasting approaches for CVD prevalence, CVD mortality, and HTN-attributable CVD deaths, and the propagation of uncertainty from data sources to outcomes.

#### Data sources

Three age-standardized inputs (CVD prevalence, CVD mortality rates, and HTN-attributable CVD deaths) were obtained from the GBD (20), covering annual estimates from 2010 – 2021. CVD prevalence was extracted as age-standardized percent, expressed as the proportion of the total population living with CVD (Supplementary Figure S3). CVD mortality rates were extracted as age-standardized rates per 100,000 person-years and converted to annual CVD death probabilities expressed as a percentage of the total population (÷ 100,000 × 100) (Supplementary Figure S4). HTN-attributable CVD deaths were extracted as the age-standardized proportion of CVD deaths attributable to high systolic blood pressure; the denominator for this metric is total CVD deaths within each sex stratum — not the total population — and is therefore not directly comparable to CVD prevalence or mortality rate estimates (Supplementary Figure S5). For all three inputs, no additional statistical manipulation was applied beyond deriving standard deviations from the reported GBD 95% uncertainty intervals using SD = (upper − lower) ÷ 3.92, which were propagated through Monte Carlo simulation. WHO STEPwise (STEPS) risk factor surveys (21) conducted in 2014 and 2024 were used for contextual validation of HTN prevalence trends but were not used as primary inputs to the modeling because they provide only two time points and were consistent with GBD estimates. HIV prevalence trajectories, stratified by age and sex, were derived from calibrated EMOD-HIV model outputs.

Relative risk (RR) estimates comparing PLHIV with HIV-negative individuals were extracted from published literature (1,12,34,35) and are summarized in Supplementary Tables S1 and S2. For HTN-associated CVD, studies commonly reported hazard ratios (HRs) by HIV status rather than direct comparative relative risks (RRs) (13). We converted HRs to approximate RRs using Equation 1 (36). Although this formula was originally derived for odds ratio-to-RR conversion, it is appropriate here because when annual baseline risks are small — as is the case in this study (≤0.4% per 100,000 population in HIV-negative adults; Supplementary Table S1) — HRs, odds ratios, and RRs are numerically similar, allowing the OR-based correction to be applied to HRs with minimal error (37).

(1) $$RR\approx \frac{HR}{\left(1-P_{0}\right)+\left(P_{0}xHR\right)}$$

where *P*_0_ represents the baseline risk of the outcome among HIV-negative adults, estimated from GBD 2021 age-standardized CVD mortality rates for Eswatini. Because GBD reports rates per 100,000 population rather than cumulative risks, *P*_0_ was derived under the exponential survival assumption using Equation 2 (38):

(2) $$P_{0}=1-e^{-r_{0}\cdot t}$$

where *r*_0_ is the GBD age-standardized HTN-attributable CVD mortality rate (per 100,000 population, converted to a proportion) used solely to derive the baseline risk *P*_0_ required for the HR-to-RR conversion; general (unstratified) CVD mortality is modeled separately as a distinct forecast outcome. *t* is the follow-up duration, aligned with the period over which the HRs were reported. A step-by-step description of the HR-to-RR conversion is provided in the Supplementary methods (Conversion of HRs to RRs), with intermediate quantities summarized in Supplementary Table S1. Because published studies (34) reported HRs separately for HIV-positive and HIV-negative adults, each expressed relative to a common external reference group rather than relative to each other, neither HR directly quantifies the excess CVD risk in PLHIV compared with HIV-negative adults. We therefore first applied Equation 1 to convert each group-specific HR into an approximate cumulative risk, then obtained the RR of HTN-associated CVD comparing PLHIV with HIV-negative adults as the ratio of these two estimated risks (Supplementary Table S2). For general CVD, RRs directly comparing PLHIV with HIV-negative adults were available from prior studies (12,35), reflecting a broader evidence base for overall CVD outcomes; by contrast, HTN-associated CVD studies typically reported hazard ratios stratified by HIV status rather than direct comparative RRs, necessitating the conversion approach described above.

#### Uncertainty in data sources

GBD estimates include uncertainty from data limitations and model specification and are reported with 95% uncertainty intervals (20). STEPS survey estimates reflect sampling variability and measurement error and are reported with survey-based confidence intervals (21). Published relative risk estimates include reported 95% confidence intervals(12,34,35). These uncertainties were propagated through the analysis using probabilistic sensitivity analysis, as described below.

#### Estimating and Forecasting CVD Prevalence

We estimated and projected cardiovascular disease (CVD) prevalence among PLHIV and HIV-negative adults through 2045 using a multi-step modeling framework (Supplementary Methods). General population age-standardized CVD prevalence from the GBD 2010–2021 dataset (Supplementary Figure S3) was decomposed into HIV-negative and PLHIV subgroups using HIV prevalence trajectories from EMOD-HIV and relative risk (RR) estimates from published literature (Supplementary Table S2) comparing PLHIV with HIV-negative adults. CVD prevalence among HIV-negative adults was calculated as:

(3) $$p_{HIV-}(t)=\frac{p_{\text{Total}}(t)}{\left[h(t)\times RR(t)]+[1-h(t)\right]}$$

where *p*_*Total*_*(t)* denotes the general population CVD prevalence, *h*(*t*) is the HIV prevalence in year *t*, and *RR*(*t*) is the relative risk of CVD among PLHIV compared with HIV-negative adults. Corresponding CVD prevalence among PLHIV was then obtained by applying the same relative risk to the decomposed HIV-negative estimates:

(4) $$p_{\text{PLHIV}}(t)=RR(t)\times p_{HIV-}(t)$$

These reconstructed time series (2010–2021) represent the empirical baseline derived from GBD, EMOD-HIV, and literature inputs. They served as the basis for forecasting future trends, with uncertainty propagated through Equations (3) and (4) as described in the Uncertainty Analysis section.

To project CVD prevalence beyond 2021, we fitted a generalized additive model (GAM) (39,40) with a logistic link and penalized splines (41), to the HIV-negative prevalence series. The logistic link constrains predictions between 0 and 1, while penalized splines capture smooth, nonlinear temporal trends. The model was specified as:

(5) $$p_{HIV-}(t)=\frac{1}{1+e^{-(\alpha +s(t))}}$$

where *p*_*HIV*−_(*t*) is the predicted HIV-negative CVD prevalence in year *t*, *α* is the intercept, and *s*(*t*) is a smooth function of time estimated using penalized splines. The fitted model was then extrapolated from 2022 to 2045, assuming that long-term changes continue smoothly along the observed temporal trajectory.

Forecasted CVD prevalence among PLHIV was subsequently derived by applying the scenario-specific relative risk *RR*(*t*) to the corresponding HIV-negative baseline for each year, maintaining internal consistency across scenarios and time periods:

(6) $$p_{\text{PLHIV}}(t)=RR(t)\times p_{HIV-}(t)$$

Logistic regression (42,43) with linear time was used as a sensitivity analysis.

#### Estimating and Forecasting CVD Mortality

Estimation and forecasting of CVD mortality followed the same modeling framework used for CVD prevalence, differing only in the choice of input data. Age-standardized CVD mortality rates (deaths per 100,000 person-years; denominator: total population) were obtained from the GBD study and converted to annual CVD death risks (probability of dying from CVD in a given year, expressed as a proportion of the total population) using an exponential survival assumption. These risks (Supplementary Figure S4) were then decomposed into HIV-negative and PLHIV strata using HIV prevalence trajectories from EMOD-HIV and scenario-specific relative risks. Reconstructed HIV-stratified CVD mortality time series for 2010–2021 served as the empirical baseline for forecasting. Future trends were projected by fitting generalized additive models with a logistic link to the HIV-negative mortality series and extrapolating through 2045, with mortality among PLHIV derived by applying the corresponding relative risks. Uncertainty was propagated analogously to the CVD prevalence analysis, as described in the Uncertainty Analysis section.

#### Estimating and Forecasting Proportion of CVD deaths Attributable to Hypertension

Forecasting of CVD deaths attributable to hypertension used the same approach, with inputs drawn from GBD estimates of HTN-attributable CVD mortality (Supplementary Figure S5). Age-standardized proportions of CVD deaths attributable to elevated systolic blood pressure (denominator: total CVD deaths within each HIV-status and sex stratum, not the total population) were reconstructed for HIV-negative and PLHIV populations using HIV prevalence trajectories and relative risk scenarios. These reconstructed series formed the baseline for projecting future trends through 2045 using generalized additive models, with scenario-specific relative risks governing divergence between PLHIV and HIV-negative adults. Uncertainty was propagated from GBD attribution estimates, HIV prevalence trajectories, and relative risks using the same probabilistic framework applied in the prevalence and mortality analyses.

#### Propagation of Uncertainty from Data Sources to Outcomes

Uncertainty in the HIV-negative forecasts was captured by obtaining 1,000 draws from the estimated covariance structure of the GAM penalized spline coefficients (assumed to follow a multivariate normal distribution) and conducting independent fits of the GAM for each draw, propagating variance from both the spline fit and smoothing penalty through the joint covariance matrix of the penalized spline coefficients, which preserves their non-independence. For PLHIV, uncertainty reflected both the GAM-derived variability in the HIV-negative baseline and uncertainty in RRs, which was incorporated by treating published point estimates and 95% confidence intervals as log-normal probability distributions (Supplementary Table S2) (44). For each parametric Monte Carlo simulation (45) iteration, RR values were sampled and applied to HIV-negative prevalence to generate corresponding estimates for PLHIV. A total of 1000 iterations yielded distributions of possible trajectories; the mean was reported as the central estimate, with 95% uncertainty intervals from the 2.5th–97.5th percentiles.

#### Scenarios

Five scenarios were explored. Scenario 1 forecasted general CVD burden among PLHIV and HIV-negative individuals using a published RR of 1.6 (1.3–2.0) (35), applied to age-standardized CVD prevalence (proportion of the total population living with CVD; denominator: total population). This scenario establishes the baseline magnitude of HIV-associated excess CVD prevalence under the assumption that the RR remains constant over the projection period. Scenario 2 forecasted general CVD mortality risk using the same RR of 1.6 (1.3–2.0) (31), applied to age-standardized CVD mortality rates converted to annual CVD death probabilities expressed as a percentage of the total population (÷ 100,000 × 100; denominator: total population). This scenario establishes the baseline magnitude of HIV-associated excess CVD mortality under the same constant-RR assumption, providing a mortality analogue to Scenario 1. Scenarios 3–5 focused on HTN-associated CVD, which accounts for approximately 55–65% of CVD cases globally and ~60% of CVD deaths in Eswatini (15,46). Sex-specific RRs were used in Scenarios 3–5 (Supplementary Table S3).

In scenario 3, RRs remained unchanged throughout the projection period – representing a counterfactual of stable relative risk – using estimates from Siddiqui et al. converted using Equation 1 and summarized in Supplementary Table S2. In scenario 4, risks were stable from 2010 through 2021, then increased in 2022 following Eswatini’s national DTG rollout in 2021 (16,47), and remained constant thereafter (women 1.4 [1.25–1.50], men 1.2 [1.10–1.30], both 1.29 [1.18–1.40]). The increase reflects DTG-associated weight gain, more pronounced among women, emerging with an approximate one-year lag (9–11). Relative-risk increases of approximately 18% in women and 14% in men were derived by translating DTG-related weight gain into BMI changes using mean adult heights from WHO STEPS Eswatini surveys (15,48), then applying published BMI-CVD risk associations (10,49,50), with detailed calculations provided in the Supplementary Information. In scenario 5, risks rose progressively from 2010, anchored to the lowest early-ART-era estimates (51) (women 1.05 [1.00–1.10], men 1.05 [1.00–1.10], both 1.1 [1.05–1.15]) and reaching target RRs by 2045 (women 1.43 [1.30–1.55], men 1.25 [1.15–1.35], both 1.33 [1.22–1.45]), reflecting the multiplicative influence of DTG-associated metabolic changes, survival-driven aging of the PLHIV population as ART scale-up continues to extend life expectancy and shift age structure (3,15), and secular increases in adiposity and hypertension across the SADC region (1,15,52), with full decomposition provided in Supplementary Note S3.

### Sensitivity analyses

Sensitivity analyses were conducted to assess the robustness of projected CVD outcomes to key modeling assumptions. First, sensitivity to the choice of extrapolation model for HIV-negative CVD outcomes was evaluated by fitting a logistic regression with a linear time trend as an alternative to the generalized additive model (GAM) and comparing resulting projections across all five scenarios; this also assessed whether the declining trend observed in HIV-negative CVD under the GAM - attributable to the downward trajectory in GBD-derived inputs over 2010–2021 - was robust to alternative model specifications. Second, uncertainty in relative risk (RR) assumptions was explored probabilistically through Monte Carlo sampling of RR distributions across all scenarios, rather than fixed point estimates. Published RR point estimates and 95% confidence intervals were treated as log-normal probability distributions (Supplementary Table S2), from which RR values were sampled and applied to HIV-negative prevalence during each simulation iteration. This approach assessed sensitivity to the magnitude of HIV- and ART-associated excess CVD risk in both the general CVD scenarios (Scenarios 1 and 2) and the HTN-associated CVD scenarios (Scenarios 3–5). Finally, projections were evaluated over alternative analytic horizons (5- and 10-year periods) in addition to the full 2010–2045 horizon to assess the stability of conclusions over shorter, policy-relevant time frames. Uncertainty in HIV prevalence trajectories from EMOD-HIV was not formally propagated through the CVD decomposition and is acknowledged as a limitation.

#### Model implementation

The overall analytical workflow is shown in Supplementary Figure S3. All analyses were conducted in Python 3. EMOD-HIV simulations generated age-and sex-stratified HIV prevalence trajectories, which were used to decompose GBD-derived CVD estimates into PLHIV and HIV-negative subgroups using the RR decomposition framework described above. Three GBD inputs were used: age-standardized CVD prevalence (Scenario 1), age-standardized CVD mortality rates converted to annual death probabilities (Scenario 2), and the age-standardized proportion of CVD deaths attributable to high systolic blood pressure (Scenarios 3–5). For each input, a logistic generalized additive model (GAM) was fitted to the 2010–2021 GBD time series and extrapolated to 2045. Scenarios 1 and 2 were analyzed using both sexes combined, whereas Scenarios 3–5 used sex-stratified analyses with sex-specific relative risks. Uncertainty was propagated through Monte Carlo simulation (N = 1,000 draws) by sampling RR values from log-normal distributions defined by published 95% confidence intervals, refitting the GAM for each draw, and computing 95% uncertainty intervals from the resulting distribution of projected outcomes.

**Figure 1.**
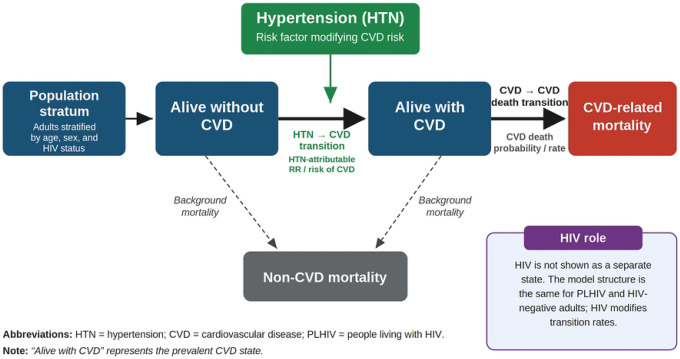
Conceptual state-transition model of CVD progression and mortality. Adults stratified by age, sex, and HIV status transition from alive without CVD to alive with CVD, and from alive with CVD to CVD-related mortality. Hypertension modifies transition to CVD, whereas non-CVD mortality acts as a competing risk from both alive states. HIV is modeled as a modifier of transition rates rather than as a separate state.

The conceptual CVD state-transition structure underlying these projections is illustrated in Figure 1. Adults were stratified by age, sex, and HIV status and could transition from alive without CVD to alive with CVD, with hypertension modeled as a risk factor modifying the transition to CVD. Individuals in the alive with CVD state could subsequently transition to CVD-related mortality, while non-CVD mortality was included as a competing risk from both alive states. HIV was not represented as a separate disease state; instead, the same model structure was applied to PLHIV and HIV-negative adults, with HIV modifying transition rates.

## Results

Across all scenarios, PLHIV exhibited higher CVD prevalence and a greater proportion of CVD deaths attributable to hypertension than HIV-negative adults, with the highest burden estimated among women. Consistent with the analytic framework described in the Methods, Scenario 1 characterizes baseline excess CVD burden using age-standardized prevalence (proportion of the total population living with CVD; denominator: total population) prevalence. Scenario 2 reports the general CVD mortality rate (annual probability of dying from CVD; denominator: total population). Scenarios 3–5 report the proportion of CVD deaths attributable to elevated systolic blood pressure (denominator: total CVD deaths within each HIV-status and sex stratum, not the total population), reflecting hypertension-mediated pathways rather than a population-level mortality rate.

### Scenario 1: General cardiovascular disease (constant RR)

**Figure 2.**
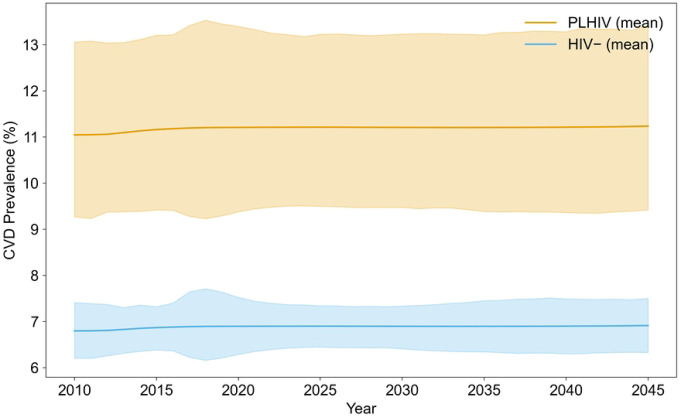
Modeled cardiovascular disease (CVD) prevalence by HIV status. Age-standardized CVD prevalence (%) with 95% uncertainty intervals under a constant relative-risk (RR = 1.6[1.3–2.0]) assumption. Projections show persistently higher CVD prevalence among PLHIV (11.0%, 95% UI 9.0–13.5%) compared with HIV-negative adults (6.8%, 95% UI 6.0–7.8%), with little change over time.

In 2025, the model estimated an age-standardized CVD prevalence of 11.0% (95% UI: 9.0–13.5%) among PLHIV and 6.8% (95% UI: 6.0–7.8%) among HIV-negative adults. Prevalence remained stable through 2045 (PLHIV: 10.9–11.2%; HIV-negative: 6.7–6.9%), maintaining an approximately 4.2 percentage-point difference between HIV-positive and HIV-negative adults, with non-overlapping uncertainty intervals (Figure 2).

### Scenario 2: General CVD mortality (constant RR)

**Figure 3.**
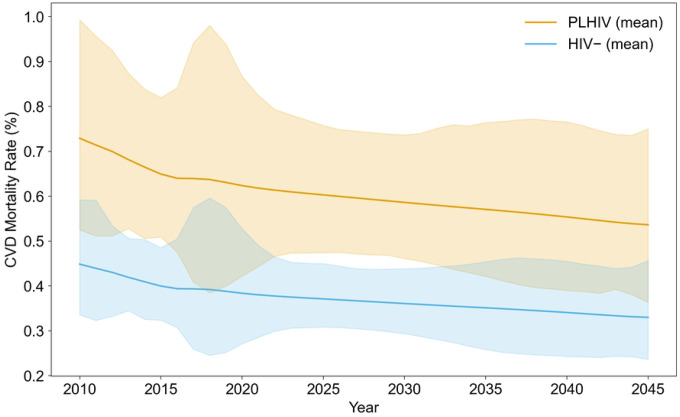
Modeled general CVD mortality risk by HIV status. Age-standardized annual probability of dying from CVD (%) with 95% uncertainty intervals under a constant relative-risk (RR = 1.6 [1.3–2.0]) assumption. PLHIV consistently exhibited higher CVD mortality risk than HIV-negative adults (0.60% vs 0.37% in 2025), with a modest declining trend reflecting GBD-derived mortality inputs and largely non-overlapping mean estimates throughout the projection horizon.

In 2025, the model estimated an age-standardized CVD mortality rate of 0.60% (95% UI: 0.47–0.76%) among PLHIV and 0.37% (95% UI: 0.31–0.45%) among HIV-negative adults. CVD mortality risk declined modestly through 2045, reflecting the downward trend in GBD-derived mortality inputs, reaching an estimated 0.54% (95% UI: 0.36–0.75%) among PLHIV and 0.33% (95% UI: 0.24–0.46%) among HIV-negative adults. PLHIV consistently exhibited higher CVD mortality risk than HIV-negative adults throughout the projection horizon, with 0.23 percentage points in 2025, narrowing slightly to 0.21 percentage points by 2045, with largely non-overlapping uncertainty intervals (Figure 3).

### Scenario 3: Hypertension-associated CVD (constant RR)

**Figure 4.**
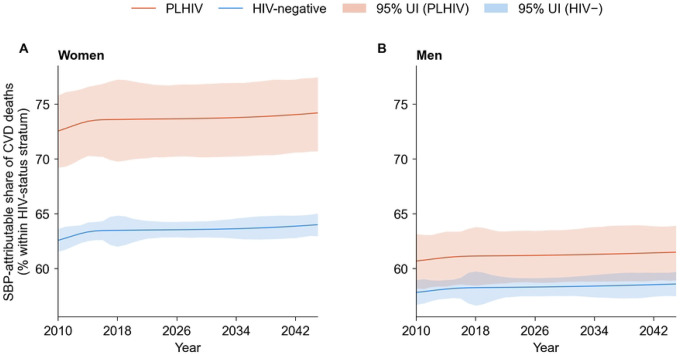
Proportion of CVD deaths attributable to high systolic blood pressure (SBP) under constant-RR assumptions. Estimated percentage of CVD deaths due to hypertension among PLHIV and HIV-negative adults, 2025–2045. The y-axis represents the proportion of total CVD deaths (denominator: all CVD deaths within each HIV-status and sex stratum) attributable to elevated SBP; it does not represent a population-level mortality rate. Under constant relative-risk assumptions, estimates change minimally over time, with consistently higher attributable fractions among PLHIV compared with HIV-negative adults.

Under a constant relative risk for hypertension-associated CVD, the proportion of CVD deaths attributable to elevated systolic blood pressure (SBP) (expressed as a percentage of total CVD deaths; denominator: all CVD deaths within each HIV-status and sex stratum) remained consistently higher among PLHIV than HIV-negative adults. In 2025, the proportion of CVD deaths attributable to elevated SBP was 73% (95% UI: 70–76%) among female PLHIV and 61% (95% UI: 58–64%) among male PLHIV, compared with 64% (95% UI: 61–66%) and 58% (95% UI: 55–60%) among HIV-negative females and males, respectively. By 2045, proportions increased modestly among PLHIV (female: 75% [95% UI: 72–78%]; male: 62% [95% UI: 59–65%]), while HIV-negative adults remained stable (Figure 4)

### Scenario 4: Hypertension-associated CVD (RR increase post-DTG)

**Figure 5.**
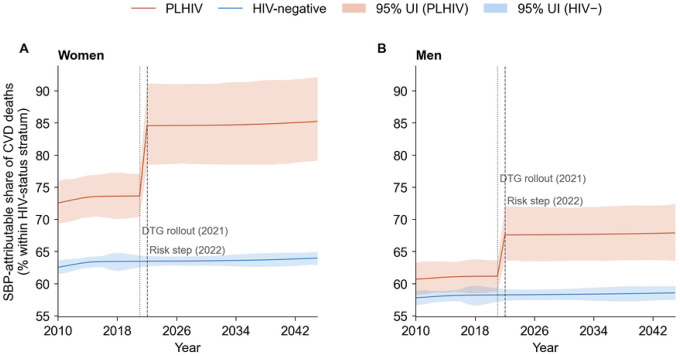
Hypertension-associated CVD mortality under post-DTG step-increase scenario. Proportion of all CVD deaths attributable to elevated systolic blood pressure (SBP) among PLHIV and HIV-negative adults. The denominator is total CVD deaths within each sex and HIV-status stratum. Relative risks increase over a one-year transition period following Eswatini’s 2021 dolutegravir rollout, reflecting staggered treatment uptake and delayed manifestation of DTG-associated metabolic effects.

This scenario incorporated a stepwise increase in RR beginning in 2022, following Eswatini’s national rollout of DTG in 2021. Prior to 2021, the proportion of all CVD deaths attributable to high SBP were 73% (70–76%) among female PLHIV and 61% (58–64%) among male PLHIV, compared with 64% (61–66%) and 58% (55–60%) among their HIV-negative counterparts. Following the DTG rollout, a one-year-lagged transition in relative risk produced a sharp rise of approximately 12 percentage points in women (73 → 85%) and 9 points in men (61 → 70%) between 2021 and 2022. Thereafter, proportions remained largely stable through 2045. By 2045, estimates reached 85% (95% UI: 82–88%) among female PLHIV and 70% (95% UI: 67–73%) among male PLHIV, while corresponding values for HIV-negative adults remained unchanged (64% [95% UI 61–66%] in females; 58% [95% UI 55–60%] in males) (Figure 5).

### Scenario 5: Hypertension-Associated CVD (Gradual Ramp 2010–2045)

**Figure 6.**
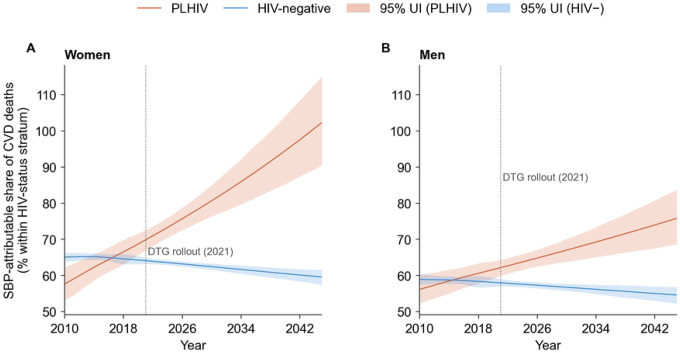
Gradual increase in hypertension-associated CVD burden. Relative risks increase log-linearly over time, reflecting cumulative effects of ART, aging, and secular hypertension trends. By 2045, female PLHIV reach 85% (95% UI 82–88%) and male PLHIV 67% (64–70%) of CVD deaths attributable to hypertension, widening the gap with HIV-negative adults.

This scenario modeled a progressive, log-linear increase in HIV-associated RR from 2010 to 2045, representing cumulative influence of ART scale-up, survival-driven aging, and secular increases in hypertension and adiposity among PLHIV. At baseline (2010), the proportions of CVD deaths attributable to high SBP were similar between HIV-positive and HIV-negative adults, approximately 63–65% in women and 55–60% in men. By 2025, proportions had risen to 78% (95% UI 74–81%) among female PLHIV and 64% (95% UI 61–67%) among male PLHIV, compared with 66% (95% UI 63–68%) in HIV-negative females and 58% (95% UI 55–60%) in HIV-negative males. Notably, by this time, the burden among male PLHIV overtook that of HIV-negative females. Gradual divergence continued over the projection horizon, with 2045 estimates reaching 85% (95% UI 82–88%) among female PLHIV and 67% (95% UI 64–70%) among male PLHIV, compared with 60% and 55% among HIV-negative women and men, respectively (Figure 6)

### Sensitivity analyses

Projected CVD outcomes were robust to alternative extrapolation assumptions across all five scenarios. Replacing the generalized additive model with a logistic regression including a linear time trend produced qualitatively similar trajectories for CVD prevalence, CVD mortality and HTN-attributable CVD deaths and did not alter the direction or relative magnitude of differences by HIV status. Differences in absolute estimates were modest and did not affect conclusions regarding the projected CVD burden (Supplementary Figures S6, S8–S14). Results were similarly stable under probabilistic sampling of relative-risk assumptions and when evaluated over shorter analytic horizons (5- and 10-year periods) (Supplementary Table S4).

## Discussion

In this modeling analysis, we projected age-standardized cardiovascular disease (CVD) prevalence, annual CVD mortality risk, and the proportion of CVD mortality attributable to hypertension (HTN) among people living with HIV (PLHIV) in Eswatini through 2045. Across all scenarios, PLHIV consistently exhibited higher CVD prevalence and a greater proportion of CVD mortality attributable to HTN than HIV-negative adults, with particularly elevated risks among women. These findings align with a growing body of evidence showing that HIV infection and antiretroviral therapy (ART) contribute to increased cardiometabolic vulnerability through inflammatory, metabolic, and treatment-related pathways (53–55). While the absolute magnitude of differences varied across the four scenarios, the direction and persistence of disparities were robust, underscoring the need to integrate CVD prevention and management within HIV service delivery platforms.

Under the constant-risk scenarios, the gap in CVD prevalence between PLHIV and HIV-negative adults remained stable, suggesting that if current risk profiles do not worsen, the excess CVD burden in PLHIV may persist but not accelerate. However, the step-change and gradual-ramp scenarios indicated substantial potential for widening disparities, particularly under conditions reflecting the metabolic effects associated with dolutegravir (DTG) and ongoing increases in hypertension and adiposity (10,56,57). The stepwise increase in hypertension-attributable CVD following Eswatini’s 2021 DTG rollout mirrors cohort evidence showing clinically meaningful weight gain within 6–18 months of DTG initiation (9–11,56,58), which in the model increases HTN prevalence and, in turn, the proportion of CVD deaths attributable to high blood pressure. The progressive, log-linear divergence observed in the ramp scenario is consistent with longitudinal data demonstrating aging of the HIV-positive population, and secular increases in obesity and hypertension across the Southern African region (1,7,59), which are represented in the model through gradually increasing relative risks rather than explicit simulation of these processes. Together, these mechanisms suggest a sustained rise in CVD burden among PLHIV in the absence of proactive mitigation. In all projections, women with HIV exhibited the highest CVD burden, reflecting sex-specific vulnerability to ART-associated weight gain and higher baseline hypertension prevalence (28,29,43), which in the model translate into higher relative risks for hypertension-associated CVD among women compared with men.

Eswatini has already made substantial progress in integrating cardiometabolic risk screening and treatment within HIV care, including routine blood pressure screening, diabetes and lipid testing, lifestyle counseling, and, in some facilities, hypertension management using simplified clinical algorithms (15,60). Our results reinforce the importance of sustaining and scaling these integrated services as the population of people living with HIV ages and ART-related metabolic risks evolve. The modeling provides quantitative evidence of the potential rise in cardiovascular disease burden over the next two decades, highlighting the need for continued investment in integrated HIV-CVD prevention and treatment. These forecasts can help guide programmatic prioritization by identifying where scale-up of hypertension screening, CVD risk assessment, and treatment within ART clinics would yield the greatest long-term health impact. Nurse-led, decentralized hypertension management models are currently being piloted in Eswatini and have demonstrated feasibility; these approaches could be leveraged to achieve high coverage and blood pressure control even in resource-limited settings (60). The pronounced divergence observed among women underscores the need to strengthen sex-specific counseling and early intervention following DTG initiation, including proactive weight monitoring and management. More broadly, this analysis complements national efforts to institutionalize cardiometabolic surveillance within HIV cohorts and supports strategic planning and resource allocation under Eswatini’s National NCD and HIV strategies (15,60).

This study has several limitations. First, EMOD-HIV does not natively simulate cardiovascular disease (18), so CVD outcomes were incorporated through post-processing. As a result, potential feedback mechanisms such as whether CVD comorbidities may influence HIV-care retention, alter ART adherence, or increase mortality independently of HIV progression were not captured. While emerging observational studies suggest that chronic comorbid conditions may influence retention in care and adherence through increased care complexity and multimorbidity, the current evidence base remains insufficient to parameterize explicit CVD–HIV feedback mechanisms within the model (61,62). Second, CVD and hypertension prevalence inputs were drawn from secondary data sources (15,20,48), which are subject to sampling error, measurement variation, and methodological differences across survey rounds (21,46). However, most contributing surveys were nationally representative and based on probability samples aligned with Eswatini’s national census, supporting the validity of population-level estimates. Third, relative risk estimates were derived from published studies that varied in population characteristics, geographic setting, and follow-up duration. Although uncertainty was propagated using Monte Carlo simulation, structural sources of uncertainty such as non-linear changes in risk over time or unmeasured confounding in the source studies, were not fully captured. Fourth, the model assumed smooth temporal trends between observed survey years, which may not fully represent short-term fluctuations or external shocks, such as changes in treatment guidelines, health policy, or pandemics. Finally, long-term projections spanning two decades inherently carry uncertainty and should be interpreted as directional trends (supported by sensitivity analysis results) rather than precise predictions. In addition, the modeling assumes a simplified, mechanistic relationship in which hypertension increases CVD risk and CVD contributes to mortality through established risk-based pathways, without explicitly representing alternative causal structures, bidirectional effects, or heterogeneity in disease progression. These structural simplifications are appropriate for population-level planning but limit causal inference.

## Conclusion

Cardiovascular disease is likely to become an increasingly important contributor to morbidity and mortality among people living with HIV in Eswatini. Without targeted intervention, the excess burden borne by PLHIV, and particularly by women, is projected to persist and, under plausible metabolic and demographic trajectories, may widen substantially. Integrating hypertension screening, treatment, and CVD prevention strategies into ART delivery represents a critical opportunity to sustain the health gains achieved through HIV treatment scale-up and to advance long-term survival and well-being in high-burden settings.

## Supplementary Material

1

## Data Availability

All data used in this study were obtained from publicly available sources, including the Global Burden of Disease (GBD) study, World Health Organization STEPwise (STEPS) surveys, and published literature, as cited in the manuscript. Processed input data used in the analyses are provided in the Supplementary Methods, including GBD-derived CVD prevalence, CVD mortality, hypertension-attributable CVD mortality inputs, and relative risk estimates used in the scenario analyses. The EMOD-HIV model is publicly available as open-source software. Analysis code is available from the corresponding author upon reasonable request.
