# Supplementary material for: Projected burden of hypertension-associated cardiovascular disease in people living with HIV versus HIV-negative adults in Eswatini
Source: medRxiv. 2026 Jul 20:2026.07.17.26358301. Preprint. [Version 1] doi: 10.64898/2026.07.17.26358301 (PMC13419648; doi:10.64898/2026.07.17.26358301)
Supplement: 1 [file NIHPP2026.07.17.26358301V1-supplement-1.pdf]

medRxiv preprint doi: https://doi.org/10.64898/2026.07.17.26358301; this version posted July 20, 2026. The copyright holder for this preprint (which was not certified by peer review) is the author/funder, who has granted medRxiv a license to display the preprint in perpetuity.
It is made available under a CC-BY 4.0 International license .

Supplementary Methods

Figure S1. Observed versus modeled age- and sex-specific HIV prevalence in Eswatini across SHIMS survey years (2007, 2011, 2016, and 2021). Points and error bars represent empirical survey estimates; lines represent EMOD-HIV model outputs. In particular, the model captured the temporal shift in peak HIV prevalence toward older age groups observed in successive survey rounds, reflecting changes in survival following ART scale-up. In addition to prevalence patterns, the model reproduced key demographic trends among people living with HIV. The mean age of the HIV-positive population increased steadily over time, rising from approximately 30 years in 2000 to 38–40 years by 2030 for both females and males (Figure S2).

Figure S2. Observed and modeled mean age of people living with HIV (PLHIV) over time, by sex. Lines show model projections; markers indicate observed or survey-derived reference points where available. These trends are consistent with observed demographic aging associated with sustained ART coverage and improved survival. Together, these calibration and validation results indicate that the model adequately represents both epidemiologic and demographic dynamics relevant to downstream projections of hypertension-associated cardiovascular disease burden.

Figure S3. Age-standardized cardiovascular disease (CVD) prevalence by sex, Eswatini, 2010–2021. Annual age-standardized CVD prevalence estimates (%) from the Global Burden of Disease (GBD) 2021 study for males, females, and both sexes combined. Lines represent point estimates and shaded bands represent 95% uncertainty intervals reconstructed from published

medRxiv preprint doi: https://doi.org/10.64898/2026.07.17.26358301; this version posted July 20, 2026. The copyright holder for this preprint (which was not certified by peer review) is the author/funder, who has granted medRxiv a license to display the preprint in perpetuity.
It is made available under a CC-BY 4.0 International license .

GBD uncertainty bounds. CVD prevalence remained relatively stable over the study period, with consistently higher prevalence among males than females. Estimates are standardized to the GBD reference population.

Figure S4. Age-standardized cardiovascular disease (CVD) mortality by sex, Eswatini, 2010–2021. Annual age-standardized CVD mortality risk (%) derived from GBD 2021 mortality rates for males, females, and both sexes combined. Lines represent point estimates and shaded bands represent 95% uncertainty intervals. Mortality declined steadily throughout the study period for both sexes, while remaining consistently higher among males. Estimates are standardized to the GBD reference population and expressed as annual probability of dying from CVD.

Figure S5. Age-standardized proportion of cardiovascular disease (CVD) deaths attributable to high systolic blood pressure (hypertension), by sex, Eswatini, 2010–2021. Annual estimates of the fraction of CVD deaths attributable to elevated systolic blood pressure (%) obtained from the GBD 2021 study for males, females, and both sexes combined. Lines represent point estimates and shaded bands represent 95% uncertainty intervals. The hypertension-attributable fraction increased gradually over time and remained consistently higher among females than males. Estimates are age-standardized to the GBD reference population.

Supplementary Note S1. Conversion of HRs to RRs

For HTN-associated CVD, published studies reported hazard ratios (HRs) rather than relative risks (RRs). To integrate these estimates into our modeling framework, we converted HRs into RRs using the following steps:

medRxiv preprint doi: https://doi.org/10.64898/2026.07.17.26358301; this version posted July 20, 2026. The copyright holder for this preprint (which was not certified by peer review) is the author/funder, who has granted medRxiv a license to display the preprint in perpetuity.
It is made available under a CC-BY 4.0 International license .

Step 1. Baseline mortality rates from GBD 2021

We obtained age-standardized CVD mortality rates attributable to high systolic blood pressure (SBP) for Eswatini from the Global Burden of Disease (GBD) 2021 study. These rates were reported as deaths per 100,000 person-years:

Males: 392.4 | Females: 325.6 | Both sexes: 360.7

Step 2. Conversion to annual hazard rate (r_0)

We first expressed these rates on a per person-year basis by dividing by 100,000:

$$r_0 = \frac{\text{GBD rate (per 100,000)}}{100{,}000}$$ Example for males: 392.4 / 100,000 ≈ 0.00392 per person-year

Step 3. Conversion to cumulative baseline risk (R_0)

Because GBD reports rates (not risks), we converted r_0 into a cumulative risk over the approximate follow-up period of the HR studies (20 years), assuming exponential survival (Greenland, 1987): $R_0 = 1 - e^{-r_0 \cdot t}$

Example for males: $R_0 = 1 - e^{-(0.00392 \times 20)} \approx 0.0392$ (or 3.92% over 20 years)

Step 4. Conversion of HR to RR

We then applied the Zhang & Yu (1998) approximation, which adjusts HRs for baseline risk:

$$RR \approx \frac{HR}{(1 - R_0) + (R_0 \cdot HR)}$$

This ensures the relative risk reflects the observed baseline incidence in the unexposed group (HIV-negative adults).

medRxiv preprint doi: https://doi.org/10.64898/2026.07.17.26358301; this version posted July 20, 2026. The copyright holder for this preprint (which was not certified by peer review) is the author/funder, who has granted medRxiv a license to display the preprint in perpetuity.
It is made available under a CC-BY 4.0 International license .

Step 5. Application to sex-specific estimates

This procedure was repeated for males, females, and all-sex categories using their respective baseline risks. Converted RRs (with 95% CIs) are presented in Table S1.

Table S1. Relative risks (RRs) of HTN-associated CVD in PLHIV versus HIV-negative adults, converted from hazard ratios (HRs) using GBD baseline risks (Eswatini, 2021) by Mohammed Siddiqui et al., 2023.

HIV status	Sex	HR (95% CI)	Baseline risk P0	Converted RR (95% CI)
HIV-negative	Male	1.51 (1.38–1.64)	0.00392	1.50 (1.36–1.63)
	Female	2.01 (1.44–2.82)	0.00326	1.99 (1.42–2.79)
HIV-positive	Male	1.67 (1.46–1.92)	0.00392	1.66 (1.45–1.90)
	Female	2.25 (1.76–2.88)	0.00326	2.23 (1.74–2.85)
HIV-negative	All sexes	1.56 (1.44–1.69)	0.00361	1.55 (1.43–1.68)
HIV-positive	All sexes	1.73 (1.52–1.96)	0.00361	1.72 (1.51–1.95)

To obtain relative risks of HTN-associated CVD in PLHIV versus HIV-negative adults, the converted RRs for HIV-positive individuals were divided by the corresponding converted RRs for HIV-negative individuals (Table S2).

Table S2. Relative risks (RRs) of HTN-associated CVD in PLHIV versus HIV-negative adults, obtained by dividing converted RRs for HIV-positive individuals by corresponding values for HIV-negative individuals.

Sex	Converted RR (95% CI)	RR (95% CI)
Male	1.66 (1.45–1.90)	1.11 (1.06 - 1.16)
Female	2.23 (1.74–2.85)	1.12 (1.02 - 1.22)
All sexes	1.72 (1.51–1.95)	1.11 (1.06 - 1.16)

Table S3. Relative risks (RRs) used in CVD forecasting scenarios

Scenario	Outcome	Time pattern	Women (95% UI)	Men (95% UI)	Both sexes (95% UI)	Sources
**Scenario 1:** General CVD (constant RR)	All CVD	Constant, 2010–2045	—	—	1.60 (1.30–2.00)	Published comparative RR for PLHIV vs HIV-negative adults (31)

medRxiv preprint doi: https://doi.org/10.64898/2026.07.17.26358301; this version posted July 20, 2026. The copyright holder for this preprint (which was not certified by peer review) is the author/funder, who has granted medRxiv a license to display the preprint in perpetuity.
It is made available under a CC-BY 4.0 International license .

Scenario	Outcome	Time pattern	Women (95% UI)	Men (95% UI)	Both sexes (95% UI)	Sources
**Scenario 2:** General CVD mortality (constant RR)	All CVD	Constant, 2010–2045	—	—	1.60 (1.30–2.00)	Published comparative RR for PLHIV vs HIV-negative adults (31)
**Scenario 3:** HTN-associated CVD (constant RR)	HTN-CVD	Constant, 2010–2045	1.16 (1.10–1.22)	1.05 (1.00–1.10)	1.10 (1.05–1.15)	Siddiqui et al.; HRs converted to RRs using Eq. 1 (30,31)
**Scenario 4:** HTN-associated CVD (post-DTG step increase)	HTN-CVD	Constant through 2021 (same as Scenario 3); step increase in 2022; constant thereafter	1.16 → 1.40 (1.25–1.50)	1.05 → 1.20 (1.10–1.30)	1.10 → 1.29 (1.18–1.40)	DTG-associated weight gain translated to BMI-CVD risk; ~1-year lag post-DTG rollout (8–10, 14, 44–46)
**Scenario 5:** HTN-associated CVD (gradual ramp)	HTN-CVD	Log-linear increase, 2010–2045	1.05 → 1.43 (1.30–1.55)	1.05 → 1.25 (1.15–1.35)	1.10 → 1.33 (1.22–1.45)	ART-era anchoring + DTG, aging, secular adiposity/HTN trends (1,3,14,47,48)

Notes: Sex-specific RRs were used for sex-stratified analyses (Scenarios 3–5); pooled "both sexes" RRs were used in analyses not stratified by sex (Scenarios 1–2). In Scenario 4, pre-2022 RRs are identical to Scenario 3, with a step increase applied beginning in 2022 following Eswatini's national DTG rollout. Full derivations for Scenario 4 step changes and Scenario 5 target values are provided in Supplementary Note S2 and S3.

RR = relative risk; UI = uncertainty interval; HTN = hypertension; CVD = cardiovascular disease; PLHIV = people living with HIV; DTG = dolutegravir; BMI = body mass index.

858 **Table S4. Projected CVD outcomes by HIV status across analytic horizons — Sensitivity**
859 **Analysis 2**

Scenario	Analytic horizon	Year	PLHIV (%)	HIV-negative (%)	Difference (pp)
**Scenario 1: General CVD prevalence (both sexes)**					
Metric: CVD prevalence (%)					
	5-year (2025–2030)	2030	11.21	6.90	**4.31**
	10-year (2025–2035)	2035	11.21	6.90	**4.31**
	20-year (2025–2045)	2045	11.23	6.91	**4.32**

medRxiv preprint doi: https://doi.org/10.64898/2026.07.17.26358301; this version posted July 20, 2026. The copyright holder for this preprint (which was not certified by peer review) is the author/funder, who has granted medRxiv a license to display the preprint in perpetuity.
It is made available under a CC-BY 4.0 International license .

Scenario	Analytic horizon	Year	PLHIV (%)	HIV-negative (%)	Difference (pp)
**Scenario 2: General CVD mortality (both sexes)**					
Metric: CVD mortality rate (%)					
	5-year (2025–2030)	2030	0.59	0.36	**0.23**
	10-year (2025–2035)	2035	0.57	0.35	**0.22**
	20-year (2025–2045)	2045	0.54	0.33	**0.21**
**Scenario 3: HTN-attributable CVD deaths, constant RR (both sexes)**					
Metric: SBP-attributable CVD deaths (% within HIV-status stratum)					
	5-year (2025–2030)	2030	70.43	60.75	**9.68**
	10-year (2025–2035)	2035	70.54	60.85	**9.70**
	20-year (2025–2045)	2045	70.91	61.17	**9.75**
**Scenario 4: HTN-attributable CVD deaths, step RR post-DTG 2022 (both sexes)**					
Metric: SBP-attributable CVD deaths (% within HIV-status stratum)					
	5-year (2025–2030)	2030	80.29	58.65	**21.64**
	10-year (2025–2035)	2035	80.40	58.73	**21.67**
	20-year (2025–2045)	2045	80.84	59.05	**21.79**
**Scenario 5: HTN-attributable CVD deaths, gradual ramp RR (both sexes)**					
Metric: SBP-attributable CVD deaths (% within HIV-status stratum)					
	5-year (2025–2030)	2030	76.74	59.41	**17.34**
	10-year (2025–2035)	2035	82.70	58.24	**24.46**
	20-year (2025–2045)	2045	95.85	55.85	**40.00**

pp = percentage points. Projections use a logistic generalized additive model (GAM) fitted to GBD 2010–2021 data and extrapolated forward. Horizons are anchored to 2025 as the baseline projection year. PLHIV = people living with HIV. RR = relative risk; DTG = dolutegravir; SBP = systolic blood pressure.

Supplementary Note S2. How DTG-associated weight gain was translated into CVD relative risks

Step 1. Excess weight gain with DTG

Randomized trials show greater weight gain with dolutegravir (DTG) compared with efavirenz (EFV): participants on DTG gained approximately 3.5 kg more than those on EFV at 192 weeks, and women receiving DTG/TAF gained about 4.8 kg more than those on EFV at 96 weeks (29).

medRxiv preprint doi: https://doi.org/10.64898/2026.07.17.26358301; this version posted July 20, 2026. The copyright holder for this preprint (which was not certified by peer review) is the author/funder, who has granted medRxiv a license to display the preprint in perpetuity.
It is made available under a CC-BY 4.0 International license .

Step 2. Converting weight gain to BMI increase

Excess weight gain (ΔW) was converted to a change in body mass index (BMI) using: $\Delta BMI = \frac{\Delta W}{H^2}$, where H is height (m). Adult heights were taken from the WHO STEPS Eswatini survey (mean 1.58 m for women and 1.68 m for men) (44). Using these values, a 3.5 kg excess gain corresponds to $\Delta BMI \approx 1.24\ for\ men\ and\ 1.40\ for\ women$, while a 4.8 kg gain corresponds to $\Delta BMI \approx 1.70$ for men and 1.92 for women.

Step 3. Mapping ΔBMI to CVD relative risk

From prior studies (46,60), vascular mortality risk increases by about 40% for every 5 kg/m² increase in BMI. To translate this into smaller BMI changes, we assumed the relationship is log-linear, that is, each 1-unit increase in BMI multiplies risk by the same factor.

- **Step 1:** From the published finding (46): +5 BMI → relative risk (RR) = 1.40.
- **Step 2:** We spread this evenly across 5 units by taking the fifth root of 1.40: R $R_{\text{per 1 BMI}} = 1.40^{1/5} \approx 1.069$, meaning each 1-unit increase in BMI raises CVD risk by ~6.9%.
- **Step 3:** For any change in BMI (ΔBMI), we raised 1.069 to the power of ΔBMI: R $R_{\text{multiplier}} = 1.069^{\Delta BMI}$. Applying these to our sex-specific ΔBMI values yields:

  - 3.5 kg excess gain
    - Men: $\Delta BMI = 1.24 \Rightarrow RR \approx 1.086$ (+8.6%)
    - Women: $\Delta BMI = 1.40 \Rightarrow RR \approx 1.098$ (+9.8%)
  - 4.8 kg excess gain
    - Men: $\Delta BMI = 1.70 \Rightarrow RR \approx 1.120$ (+12.0%)
    - Women: $\Delta BMI = 1.92 \Rightarrow RR \approx 1.137$ (+13.7%)

medRxiv preprint doi: https://doi.org/10.64898/2026.07.17.26358301; this version posted July 20, 2026. The copyright holder for this preprint (which was not certified by peer review) is the author/funder, who has granted medRxiv a license to display the preprint in perpetuity.
It is made available under a CC-BY 4.0 International license .

This corresponds ~9–10% higher CVD risk for 3.5 kg excess gain and ~12–14% for a 4.8 kg gain, with larger effects in women than men. In our scenario analyses, we modeled slightly larger step increases (~18% in women and ~14% in men) to account for additional DTG-associated metabolic effects beyond BMI alone.

Supplementary Note S3. Decomposition of RR trajectories in the gradual-ramp scenario (scenario 5)

Scenario 5 represents a gradual, long-term increase in hypertension-associated cardiovascular disease (HTN-CVD) risk among PLHIV relative to HIV-negative adults between 2010 and 2045. Rather than assuming an abrupt change in RR, this scenario reflects the combined influence of several slow-moving, compounding drivers that elevate relative risk over time. These processes were represented as multiplicative calibration components, including dolutegravir (DTG)-associated metabolic effects, survival-driven aging of the PLHIV population, and secular increases in adiposity and hypertension in the background population, each contributing to the widening PLHIV–HIV-negative gap.

These multipliers were not modeled as independent causal estimates and were not used as uncertainty-analysis ranges. Instead, they were specified as transparent, approximate calibration components chosen to generate plausible 2045 RRs consistent with the hypothesized epidemiologic trajectory in each stratum. Table S5A summarizes the conceptual interpretation and empirical motivation for each calibration component, whereas Table S5B reports the exact sex-specific values used to ensure that the product of the baseline RR and the component multipliers matched the target 2045 RR in each stratum.

Framework

For each stratum, the target RR in 2045 was defined as:

$RR_{2045} = RR_{2010} \times M_{\mathrm{DTG}} \times M_{\mathrm{Aging}} \times M_{\mathrm{Secular}}$where:

medRxiv preprint doi: https://doi.org/10.64898/2026.07.17.26358301; this version posted July 20, 2026. The copyright holder for this preprint (which was not certified by peer review) is the author/funder, who has granted medRxiv a license to display the preprint in perpetuity.
It is made available under a CC-BY 4.0 International license .

- RR_{2010} is the baseline RR in 2010;
- M_{DTG} is the multiplier representing gradual DTG-associated metabolic effects;
- M_{Aging} is the multiplier representing survival-driven aging of the PLHIV population; and
- M_{Secular} is the multiplier representing secular increases in adiposity and hypertension in the background population.

Baseline RRs in 2010 were set to 1.05 for women, 1.05 for men, and 1.10 for the combined-sex analysis. Target RRs in 2045 were set to 1.43 for women, 1.25 for men, and 1.33 for the combined-sex analysis.

Interpretation and empirical motivation of calibration components

The three calibration components were chosen to reflect plausible long-term drivers of increasing HTN-CVD risk among PLHIV. As shown in Table S5A, M_{DTG} was motivated by evidence on DTG-associated weight gain and metabolic effects, M_{Aging} by evidence that ART scale-up extends survival and shifts the PLHIV population toward older ages, and M_{Secular} by evidence of rising obesity and hypertension in Eswatini and the broader southern African region.

medRxiv preprint doi: https://doi.org/10.64898/2026.07.17.26358301; this version posted July 20, 2026. The copyright holder for this preprint (which was not certified by peer review) is the author/funder, who has granted medRxiv a license to display the preprint in perpetuity.
It is made available under a CC-BY 4.0 International license .

Table S5A. Literature-informed interpretation of calibration components in Scenario 5 (Gradual Ramp)

Component	Description	Empirical basis / justification	Approximate cumulative contribution by 2045
M_{DTG}	Gradual metabolic and weight-gain effects associated with dolutegravir (DTG), modeled as a smooth ramp beginning in 2021	ADVANCE and NAMSAL trials; BMI-to-CVD conversion informed by Prospective Studies Collaboration and related literature	Approximately 10% for men and 15% for women
M_{Aging}	Increased exposure to CVD risk factors as ART scale-up extends survival and shifts the PLHIV population toward older ages	Nakagawa et al., 2018; Harris et al., 2021; UNAIDS, 2013	Approximately 5%–10%
M_{Secular}	Background rise in obesity and hypertension in Eswatini and the broader southern African region	WHO STEPS Eswatini, 2024; Gona et al., 2021; Barry et al., 2025; WHO AFRO, 2024	Approximately 10%–15%

Note: Approximate contributions represent literature-informed conceptual magnitudes by 2045 and are not uncertainty-analysis ranges or independently estimated causal effects.

The approximate cumulative contributions shown in Table S5A are intended to convey the conceptual magnitude of each component by 2045; they are not uncertainty bounds and were not applied directly as ranges in the model.

Exact calibration values used to match the 2045 targets

The exact calibration values used in the model are shown in Table S5B.

Table S5B. Exact calibration values used to match the target 2045 relative risks in Scenario 5 (Gradual Ramp)

Component	Women	Men	Both sexes
RR_{2010}	1.05	1.05	1.10
M_{DTG}	1.15	1.10	1.10
M_{Aging}	1.08	1.03	1.05
M_{Secular}	1.0965	1.0507	1.0468
RR_{2045}	1.43	1.25	1.33

medRxiv preprint doi: https://doi.org/10.64898/2026.07.17.26358301; this version posted July 20, 2026. The copyright holder for this preprint (which was not certified by peer review) is the author/funder, who has granted medRxiv a license to display the preprint in perpetuity.
It is made available under a CC-BY 4.0 International license .

These values were selected jointly within each stratum so that the product $RR_{2045} = RR_{2010} \times M_{\text{DTG}} \times M_{\text{Aging}} \times M_{\text{Secular}}$ equaled the target 2045 RR exactly. These calibration values satisfy the target RRs exactly:

$$RR_{2045,women} = 1.05 \times 1.15 \times 1.08 \times 1.097 = 1.43$$

$$RR_{2045,men} = 1.05 \times 1.10 \times 1.05 \times 1.047 = 1.25$$

$$RR_{2045,both} = 1.10 \times 1.10 \times 1.05 \times 1.047 = 1.33$$

Interpretation of component multipliers

The component multipliers should be interpreted as cumulative calibration factors operating over the full 2010-2045 period. For example M_{Aging} 1.08 for women indicates an assumed 8% cumulative increase in RR by 2045 attributable to survival-driven aging of the PLHIV population, relative to the 2010 baseline. These values do not represent annual increases. Rather, they summarize the total contribution of each component to the 2045 RR endpoint.

Temporal implementation

After specifying RR_{2010} and RR_{2045}, the year-specific RR trajectory was modeled as a smooth log-linear increase from 2010 to 2045:

$$RR(t) = RR_{2010} \times \exp[\frac{\ln(RR_{2045}/RR_{2010})}{35}(t-2010)], \qquad t \in [2010, 2045]$$

This formulation yields a continuous increase in RR over time, consistent with gradual epidemiologic change rather than an abrupt intervention-induced shift (Figure S3).

Figure S6. Relative risk trajectories applied to PLHIV versus HIV-negative adults – HTN-attributable CVD Deaths, Scenarios 3-5 (2010 – 2045)

medRxiv preprint doi: https://doi.org/10.64898/2026.07.17.26358301; this version posted July 20, 2026. The copyright holder for this preprint (which was not certified by peer review) is the author/funder, who has granted medRxiv a license to display the preprint in perpetuity.
It is made available under a CC-BY 4.0 International license .

4. References for Supplementary Note S3

1. Nakagawa F et al. *The aging HIV epidemic: demographic projections and implications for care. PLoS One.*2018;13(2):e0190455.
2. Harris TG et al. *HIV and aging among adults aged ≥50 years on ART in sub-Saharan Africa. Afr J AIDS Res.*2021;20(2):115–125.
3. UNAIDS. *HIV and Aging.* Geneva: UNAIDS; 2013.
4. Gona PN et al. *Changes in body mass index, obesity, and overweight in SADC countries. BMC Public Health.*2021;21:1684.
5. WHO Regional Office for Africa. *Obesity rising in Africa – WHO analysis finds.* Brazzaville: WHO AFRO; 2024.
6. Barry M et al. *Non-communicable diseases in the WHO African Region. Sci Rep.* 2025;15:97180.
7. Kingdom of Eswatini & WHO. *Eswatini STEPS 2024 Report: Non-communicable disease risk factors.* Mbabane: Ministry of Health and WHO; 2025.
8. Forouzanfar MH et al. *Global burden of hypertension and systolic blood pressure of at least 110–115 mm Hg, 1990–2015. NEJM.* 2017;377(2):165–176.
9. Venter WF et al. *Weight gain on dolutegravir and tenofovir alafenamide: ADVANCE trial 96-week results. Lancet HIV.* 2019;6:e632–e643.

Steps: Estimating and forecasting CVD outcomes

Step 1: Decomposing total population CVD estimates into HIV-status strata.

General population age-standardized CVD outcomes were obtained from GBD 2021 annual estimates for Eswatini (2010–2021; Supplementary Table S1).

Because GBD provides total population estimates rather than HIV-stratified values, CVD outcomes among HIV-negative adults were derived using a conditional probability decomposition. The total population CVD outcome can be expressed as:

medRxiv preprint doi: https://doi.org/10.64898/2026.07.17.26358301; this version posted July 20, 2026. The copyright holder for this preprint (which was not certified by peer review) is the author/funder, who has granted medRxiv a license to display the preprint in perpetuity.
It is made available under a CC-BY 4.0 International license .

$$p_{\text{Total}}(t) = h(t) \cdot p_{\text{PLHIV}}(t) + (1 - h(t)) \cdot p_{\text{HIV}-}(t) \qquad (1)$$

where $h(t)$is the HIV prevalence in the general population at time t, derived from calibrated EMOD-HIV model outputs. Applying the scenario-specific relative risk $\text{RR}(t)$ - defined as the ratio of the CVD outcome in PLHIV relative to HIV-negative adults - gives $p_{\text{PLHIV}}(t) = \text{RR}(t) \times p_{\text{HIV}-}(t)$. Substituting and solving for $p_{\text{HIV}-}(t)$:

$$p_{\text{HIV}-}(t) = \frac{p_{\text{Total}}(t)}{h(t) \cdot \text{RR}(t) + (1 - h(t))} \qquad (2)$$

CVD outcomes among PLHIV were then recovered as:

$$p_{\text{PLHIV}}(t) = \text{RR}(t) \times p_{\text{HIV}-}(t) \qquad (3)$$

These reconstructed time series (2010–2021) represent the empirical baseline derived from GBD, EMOD-HIV, and published RR estimates (Supplementary Table S3). They served as the basis for forecasting future trends, with uncertainty propagated through Equations (2) and (3) as described in the Uncertainty Analysis section.

Step 2: Forecasting HIV-negative CVD outcomes using a generalized additive model.

To project CVD prevalence among HIV-negative adults beyond 2021, we fitted a generalized additive model (GAM) with a logistic link function to the HIV-negative series derived in Step 1:

$$\text{logit}(p_{\text{HIV}-}(t)) = \alpha + s(t) \qquad (4)$$

$$p_{\text{HIV}-}(t) = \frac{1}{1 + e^{-(\alpha + s(t))}} \qquad (5)$$

where $p_{\text{HIV}-}(t)$is the predicted HIV-negative CVD outcome in year t, $\alpha$is the intercept, and $s(t)$ is a smooth function of time estimated using penalized splines. The logistic link constrains

medRxiv preprint doi: https://doi.org/10.64898/2026.07.17.26358301; this version posted July 20, 2026. The copyright holder for this preprint (which was not certified by peer review) is the author/funder, who has granted medRxiv a license to display the preprint in perpetuity.
It is made available under a CC-BY 4.0 International license .

predicted values between 0 and 1, while penalized splines capture smooth, nonlinear temporal trends. The fitted model was extrapolated from 2022 to 2045, assuming that long-term changes continue smoothly along the observed temporal trajectory. Logistic regression with a linear time trend was used as a sensitivity analysis (38, 39).

Step 3: Forecasting CVD outcomes among PLHIV.

Forecasted CVD outcomes among PLHIV were derived by applying the scenario-specific relative risk $\mathrm{RR}(t)$to the corresponding HIV-negative forecast from Step 2 for each year:

$$p_{\mathrm{PLHIV}}(t) = \mathrm{RR}(t) \times p_{\mathrm{HIV-}}(t) \qquad (6)$$

This formulation maintains internal consistency across scenarios and time periods, ensuring that stratum-specific estimates remain anchored to the HIV-negative baseline and reflect only the scenario-specific excess CVD risk among PLHIV.

Figure S7. Analytical workflow for projecting cardiovascular disease burden among adults in Eswatini, 2025–2045. Workflow integrating EMOD-HIV outputs, GBD 2021 inputs, published relative risks, GAM-based forecasting, and Monte Carlo uncertainty quantification to project five CVD scenarios through 2045. Outcomes for PLHIV and HIV-negative adults were estimated using RR-based decomposition of GBD-derived population measures.

Figure S8. Modeled cardiovascular disease (CVD) prevalence by HIV status and sex. Age-standardized CVD prevalence (%) with 95% uncertainty intervals under a constant relative-risk (RR = 1.6[1.3-2.0]) assumption. Male PLHIV exhibited the highest prevalence (~11.7%, 95%

medRxiv preprint doi: https://doi.org/10.64898/2026.07.17.26358301; this version posted July 20, 2026. The copyright holder for this preprint (which was not certified by peer review) is the author/funder, who has granted medRxiv a license to display the preprint in perpetuity.
It is made available under a CC-BY 4.0 International license .

UI: 9.5–13.5% in 2025), followed by female PLHIV (~10.8%, 95% UI: 9.0–12.5%), male HIV-negative adults (~7.2%, 95% UI: 6.6–7.8%), and female HIV-negative adults (~6.6%, 95% UI: 6.2–7.2%), with all differences remaining stable and non-overlapping through 2045.

Note: Projected CVD prevalence was higher among male PLHIV than female PLHIV, a pattern driven by the decomposition method: because HIV prevalence is lower among men than women in Eswatini, the relative-risk adjustment concentrates excess CVD burden across a smaller PLHIV denominator, yielding higher estimated prevalence per PLHIV individual. This reflects a mathematical consequence of the decomposition framework rather than a biological difference in CVD risk by sex, and should be interpreted accordingly.

Figure S9. Modeled general CVD mortality risk by HIV status. Age-standardized annual probability of dying from CVD (%) with 95% uncertainty intervals under a constant relative-risk (RR = 1.6 [1.3–2.0]) assumption. PLHIV consistently exhibited higher CVD mortality risk than HIV-negative adults across both sexes throughout the projection horizon, with a modest declining trend reflecting GBD-derived mortality inputs.

Figure S10. Sensitivity analysis: GAM versus linear time trend extrapolation — Scenario 1 (General CVD Prevalence, Constant RR). Age-standardized CVD prevalence (%) among PLHIV and HIV-negative adults projected from 2010 to 2045 using a generalized additive model (GAM; left panel) and a logistic regression with a linear time trend (right panel). Both sexes combined; RR = 1.6 (95% CI: 1.3–2.0). Shaded ribbons represent 95% uncertainty intervals.

medRxiv preprint doi: https://doi.org/10.64898/2026.07.17.26358301; this version posted July 20, 2026. The copyright holder for this preprint (which was not certified by peer review) is the author/funder, who has granted medRxiv a license to display the preprint in perpetuity.
It is made available under a CC-BY 4.0 International license .

Figure S11. Sensitivity analysis: GAM versus linear time trend extrapolation — Scenario 2 (General CVD Mortality, Constant RR). Annual CVD death probability (%) among PLHIV and HIV-negative adults projected from 2010 to 2045 using a generalized additive model (GAM; left panel) and a logistic regression with a linear time trend (right panel). Both sexes combined; RR = 1.6 (95% CI: 1.3–2.0). Shaded ribbons represent 95% uncertainty intervals.

Figure S12. Sensitivity analysis: GAM versus linear time trend extrapolation — Scenario 3 (HTN-Attributable CVD Deaths, Constant RR). SBP-attributable share of CVD deaths (% within HIV-status stratum) among PLHIV and HIV-negative adults projected from 2010 to 2045 using a generalized additive model (GAM; left panel) and a logistic regression with a linear time trend (right panel). Both sexes combined; Shaded ribbons represent 95% uncertainty intervals.

Figure S13. Sensitivity analysis: GAM versus linear time trend extrapolation — Scenario 4 (HTN-Attributable CVD Deaths, Post-DTG Step Increase). SBP-attributable share of CVD deaths (% within HIV-status stratum) among PLHIV and HIV-negative adults projected from 2010 to 2045 using a generalized additive model (GAM; left panel) and a logistic regression with a linear time trend (right panel). Step increase in RR applied to PLHIV from 2022 following Eswatini's national dolutegravir rollout; HIV-negative adults held constant. Both sexes combined; sex-specific RRs applied. Shaded ribbons represent 95% uncertainty intervals.

Figure S14. Sensitivity analysis: GAM versus linear time trend extrapolation — Scenario 5 (HTN-Attributable CVD Deaths, Gradual Ramp). SBP-attributable share of CVD deaths (% within HIV-status stratum) among PLHIV and HIV-negative adults projected from 2010 to 2045

medRxiv preprint doi: https://doi.org/10.64898/2026.07.17.26358301; this version posted July 20, 2026. The copyright holder for this preprint (which was not certified by peer review) is the author/funder, who has granted medRxiv a license to display the preprint in perpetuity.
It is made available under a CC-BY 4.0 International license .

using a generalized additive model (GAM; left panel) and a logistic regression with a linear time trend (right panel). Log-linear RR increase from 2010 anchored to early-ART-era estimates, reaching target values by 2045; applied to PLHIV only. Both sexes combined; sex-specific RRs applied. Shaded ribbons represent 95% uncertainty intervals.
